# Adaptive temperature regulation in the little bird in winter: predictions from a stochastic dynamic programming model

**DOI:** 10.1007/s00442-017-3923-3

**Published:** 2017-08-03

**Authors:** Anders Brodin, Jan-Åke Nilsson, Andreas Nord

**Affiliations:** 1 Department of Biology, Lund University, Lund, Sweden; 2 Present Address: Department of Arctic and Marine Biology, University of Tromsø, Tromsø, Norway

**Keywords:** Hypothermia, Little bird in winter, Facultative hypothermia, Body temperature regulation, Winter fattening, Dynamic programming, Heterothermy

## Abstract

Several species of small birds are resident in boreal forests where environmental temperatures can be −20 to −30 °C, or even lower, in winter. As winter days are short, and food is scarce, winter survival is a challenge for small endothermic animals. A bird of this size will have to gain almost 10% of its lean body mass in fat every day to sustain overnight metabolism. Birds such as parids (titmice and chickadees) can use facultative hypothermia, a process in which body temperature is actively down-regulated to a specific level, to reduce heat loss and thus save energy. During cold winter nights, these birds may decrease body temperature from the normal from 42 ° down to 35 °C, or even lower in some species. However, birds are unable to move in this deep hypothermic state, making it a risky strategy if predators are around. Why, then, do small northern birds enter a potentially dangerous physiological state for a relatively small reduction in energy expenditure? We used stochastic dynamic programming to investigate this. Our model suggests that the use of nocturnal hypothermia at night is paramount in these biomes, as it would increase winter survival for a small northern bird by 58% over a winter of 100 days. Our model also explains the phenomenon known as winter fattening, and its relationship to thermoregulation, in northern birds.

**Electronic supplementary material:**

The online version of this article (doi:10.1007/s00442-017-3923-3) contains supplementary material, which is available to authorized users.

## Introduction

Small passerines that are residents at northern latitudes face a formidable energetic challenge in winter. In the boreal forest, environmental temperatures may be very low for prolonged periods, at the same time, as days are short and food availability low. Furthermore, small animals have a relatively larger surface area for heat exchange compared to their body volume than larger animals. This means that they must have a higher metabolism than large animals to maintain a stable body temperature. Small passerines such as the blue tit (*Cyanistes caeruleus*), the willow tit (*Poecile montanus*), or the black-capped chickadee (*P. atricapillus*) with body masses ranging from 10 to 13 g have a day-time body temperature of 41–43 °C in winter (e.g., Haftorn 1972; but see Lewden et al. 2014) which may be 70–80 °C higher than their environment. As they cannot forage when it is dark, they need to gain almost 10% of their lean body mass in fat every day to fuel overnight metabolism in winter (Haftorn 1992).

It may appear as if small birds should carry as large fat reserves as possible under such conditions, but small birds cannot afford to carry superfluous fat: a heavy bird may not be agile enough to escape airborne predators, such as sparrow hawks (*Accipiter* spp.) and pygmy owls (*Glaucidium* spp.) (McNamara and Houston 1990; Brodin 2007). Furthermore, to build up large fat deposits, birds must be active foragers. A bird that forages must move, and may then be more exposed to predators than a bird that rests in cover (e.g., Lima 1985). This trade-off between death from starvation and predation has made “the little bird in winter” a popular model system for behavioural ecologists, physiologists, and theoreticians (see Brodin 2007 for a review of this literature).

To minimize the amount of fat carried, energy management must be as economical as possible. One way of reducing energy expenditure is to decrease body temperature. This will lower energy expenditure in two ways; via reduced heat loss and lower metabolic demands of colder tissues. Accordingly, a 10–13 g parid may lower its night-time body temperature from 42–43 to 35 °C, and sometimes even lower, which reduces their energy expenditure considerably (Haftorn 1972; Reinertsen and Haftorn 1986) For a review of avian facultative hypothermia, see McKechnie and Lovegrove (2002).

A bird in deep hypothermia will be in a torpor-like condition, and remain motionless even when they are touched or lifted (Haftorn 1972). Even moderate reductions of body temperature may reduce reactivity and increase predation risk (Carr and Lima 2013). This has led to the assumption that hypothermia incurs a trade-off between the benefit of energy saving and the cost of increased predation risk. This trade-off probably explains why rest-phase body temperature varies on a daily basis coincident, e.g., with variation in the environment and body condition (e.g., Nord et al. 2009, 2011). Also birds that do not actively decrease their body temperatures will have 1–2 °C lower body temperatures at night as a natural part of their circadian cycle (Prinzinger et al. 1991). Birds sleeping in this condition are able to react and fly if disturbed. We are aware that there might also be trade-offs between temperature regulation and other regulatory processes. For example, small birds seem to have impaired immune function at hypothermic body temperatures (Nord et al. 2013; Sköld-Chiriac et al. 2015), and may also suffer sleep loss when in hypothermia (e.g., Mueller et al. 2012) with potential consequences for memory retention in hoarding species (Roth et al. 2010). However, we do not explore such potential costs in this model.

Facultative hypothermia appears to be an advanced adaptation to cold conditions for small birds living under boreal winter conditions. Yet, the relative energy savings from hypothermia gradually become smaller as the ambient temperature decreases (e.g., Reinertsen and Haftorn 1986; Saarela et al. 1995; Maddocks and Geiser 1997). At an ambient temperature of 20 °C, an 11.5 g parid would save 35% of its night-time energy expenditure by decreasing its body temperature by 8 ° down to 32 °C. At an ambient temperature of −20°, however, it would save only 10% (Reinertsen and Haftorn 1986), because (as body insulation is constant) heat production must increase to meet the energy costs of thermoregulation in the cold. Such seemingly modest reductions in rest-phase body temperature, and small associated energy savings, contrast sharply with data for some other bird orders, such as hummingbirds (Trochilidae). These birds may lower their body temperature as low as 7 °C during torpor bouts (e.g., Carpenter 1974; reviewed by Ruf and Geiser 2015), and save 90% or more energy relative to basal metabolism in the process (Ruf and Geiser 2015). From this perspective, the relevant question may somewhat unexpectedly become: why would small boreal birds use this dangerous strategy for a relatively small reduction in energy expenditure? As the parameter values of the energetics of small parids in winter are relatively well known, we think that a stochastic dynamic programming model is a well-suited tool to investigate this question.

Three previous dynamic programming models have focussed on night-time hypothermia in small birds (Clark and Dukas 2000; Pravosudov and Lucas 2000; Welton et al. 2002). Two of these treat hypothermia as a strategic choice of whether or not to enter maximum hypothermia at dusk (Pravosudov and Lucas 2000; Welton et al. 2002). The third model allows birds to choose the depth of hypothermia, with shallow hypothermia being less risky than deep hypothermia (Clark and Dukas 2000). In accordance with the two other models, Clark and Dukas (2000) also consider hypothermia as a choice that is made for the duration of the whole night.

Our knowledge of the regulation of body temperature during facultative hypothermia has increased considerably, since these models were published. For example, it has been shown that mourning doves *Zenaida macroura* in shallow hypothermia are still able to fly if attacked (Carr and Lima 2013). In addition, black-capped chickadees may show hypothermic body temperatures during daylight hours (Lewden et al. 2014, 2017). Finally, blue tits *Cyanistes caeruleus* may follow trajectories of shallow hypothermia during whole nights, with the depth depending on, e.g., roost site microclimate (Nord et al. 2011). In accordance with many empirical studies (e.g., Steen 1958; Haftorn 1972; Wolf and Hainesworth 1972; Reinertsen and Haftorn 1986; Nord et al. 2009, 2011), we, therefore, consider body temperature to be a variable that can be precisely regulated in the same way as body fat deposits or foraging effort. For example, birds should be able to enter or leave hypothermia anytime during the 24-h day. This means that our model is different from earlier ones, because it treats facultative hypothermia as a state variable that can be controlled by the bird on a short-term basis. We are mainly interested in energy regulation during a typical day in mid-winter, and not, for example, in seasonal responses to variation in photoperiod.

## Methods

To calculate the optimal sequence of behaviours, we used stochastic dynamic programming (e.g., Houston et al. 1988; Mangel and Clark 1988). A model of this type consists of a backward (in time) calculation of optimal behaviours using a dynamic programming algorithm, frequently followed by a forward iteration (Clark and Mangel 2000). The forward iteration is described at the end of the methods section. The parameter values are taken from data on willow tits (Tables 1, 2). This species, however, is a large-scale food-hoarder (Brodin 2005), meaning that it can optimize energy regulation in at least three interacting dimensions; body fat deposits, amount of cached food, and depth of hypothermia. As we were specifically interested in the interaction between the energy saving form hypothermia and body fat regulation, we did not include food-storing in our model. Our model animal is, thus, a non-hoarding parid with a body mass of 10–13 g, such as a blue tit. We are aware that blue tits may not be as cold-adapted as, for example, a willow tit.

**Table 1:** Baseline values for the behaviours

Behaviour	*α* (kJ)	*C*_RM_ (kJ)^a^	*λ*	*β* ^b^
1. Forage 1	80	45	0.8	2.5 × 10^−3^ × *x*_p_
2. Forage 2	60	45	0.8	1.25 × 10^−3^ × *x*_p_
3. Rest	0	45	1	2.5 × 10^−4^
4. Decrease	0	45–*y*_save_	1	5 × 10^−2^/3.7 × 10^−4^
5. Increase	0	45–*y*_save_	1	5 × 10^−2^/3.7 × 10^−4^
6. Constant hypoth	0	45–*y*_save_	1	5 × 10^−2^/3.7 × 10^−4^

The values are given per day rather than per 5-min period, since this is more intuitive and comparable with literature

^a^Metabolism when foraging is linearly mass-dependent

^b^Predation risk is an accelerating mass-dependent cost. For behaviours 4–6, the first *β* value is for day-time hypothermia, the second for night-time hypothermia

**Table 2:** Parameter values

Symbol	Parameter	Value
*X*_max_	Max body fat deposits^a^	148 kJ (4 g fat, 100 discrete steps)
*Y*_max_	Max degrees hypothermia^b^	7 °C (20 discrete steps)
*D*	Number of days in winter	100
*T*	Number of time periods in winter	288,896 (288 × 100 + 96)
*γ*	Increase energy expenditure cold weather	20%
*δ*	Reduced energy gain unsuccessful foraging	20%
*ε*	Reduced energy expenditure max hypothermia	30%
*x*_start_	Fat deposits start of forward iteration	12 kJ
*C*_WUi_	Extra warming up cost hypothermic birds	0 or 6 kJ

^a^Ceiling of the model, much more than the model ever predicts any bird to carry

^b^The unit for hypothermia in the model is % save of resting metabolism. The maximum save (30%) is translated to 7 °C to facilitate reading of the figures

We first define our dynamic programming functions as:

1 $$\begin{aligned} F_{W} \left( {x,y,t,T} \right) = {\text{the maximum probability that the bird survives}} \hfill \\ {\text{from the beginning of time period}}\;t\;{\text{until the last period of winter}} \hfill \\ T|(t) = x,Y(t) = y\;{\text{and}}\;W(t) = w, \hfill \\ \end{aligned}$$

where *X* is the amount of body fat deposits with its current value *x*, *Y* is the decrease in body temperature (as a positive number of degrees °C) with its current value *y*, and, *W* is the weather with the current condition *w*. *X* and *Y* are the state variables of the model, which the bird can affect with its decisions. *W* is an external variable that the animal cannot affect, but that it can behave in response to. To simplify the interpretation of our results and reduce the size of the model, weather has a distribution with only two conditions: good, G or bad, B. A realistic weather distribution (with *n* conditions) would need a time-dependent *n*th order Markov chain with transition probabilities for all possible combinations of body fat levels and hypothermic conditions. This would give the model an unmanageable number of dimensions (see Clark and Dukas 2000 for a discussion of this). The only difference between the two weather types is that energy expenditure is higher under bad conditions. The conditions can occur both during day-time and night-time hours. We considered good weather as the more common condition and bad weather as a deviation from this. It should be emphasized that the rationale here is not primarily to simulate realistic weather, but to create necessary unpredictability in the model.

*T* is the total number of time intervals over the whole winter, with *t* denoting the present time interval. In a model of this type, the length of the time intervals should be chosen, so they are appropriate for new foraging decisions in the modelled species. We think that it is reasonable that a small passerine, such as a parid, makes a new foraging decision every fifth minute (see the explanation of Eq. 7 for more details). Such a decision could be whether to increase foraging intensity or not, whether to rest or to forage, whether to leave the present patch of food, etc. This means that there are 288 time periods in one 24 h day, *D*, and that the model in total covers 100 × 288 periods. For practical reasons, we have added 96 extra intervals at the end of the last day of winter. The reason is that the 24-h day starts and ends at midnight. If winter would end at midnight, the bird would need to carry sufficient fat to buffer for the 4 h of night that remains before the next morning. Because weather is stochastic, and time intervals short, this would be problematic to describe mathematically. Besides, the terminal reward function is already complicated by another energy buffer; a bird that happens to be in a hypothermic state in the last period will need to carry sufficient fat to cover the warming up cost.

As the scope of the model is winter survival, the final time period *T* is defined as the last time period of winter, occurring at daybreak after the last night of winter. The survival probability of a bird, this period, is described by the following:

2 $$F(x,y,T,T) = \left\{ {\begin{array}{*{20}c} {1{\text{ if }}x \ge x_{{{\text{C(}}y )}} } \\ {0{\text{ if }}x < x_{{{\text{C(}}y )}} } \\ \end{array} } \right.,$$

where $x_{{{\text{C(}}y )}}$ is the amount of fat needed for a hypothermic bird to warm up, which is a function of the degree of hypothermia *y*. For a non-hypothermic bird, $x_{{{\text{C(}}y )}}$ will be 0. Realistically, a bird would not know beforehand precisely at what date the winter will end. Hence, the terminal reward can not only occur at *t* = *T*, but also in a time span between the first, *T*_f_, and the last possible period that winter can end, *T*. We are modelling a winter of 100 days and assume that *T*_f_ may occur any period from the start of day 80 and onwards. Such an uncertain terminal reward function may be over-cautious as the shape of this function hardly will affect behaviour in mid-winter (McNamara et al. 1990). If the probability *p* that winter ends a particular period *t* between *T*_f_ and *T* is uniform over time, it can be calculated by the following:

3 $$p_{t} = \frac{1}{(T + 1) - t}.$$

For time periods between *T*_f_ and *T*, the terminal reward, here denoted *Φ*, is analogous to (2):

4 $$\varPhi (x,y,t) = \left\{ {\begin{array}{*{20}c} {1{\text{ if }}x \ge x_{{{\text{C(}}y )}} {\text{ and }}t = 96} \\ {0{\text{ if }}x < x_{{{\text{C(}}y )}} {\text{ or }}t \ne 96} \\ \end{array} } \right.,$$

where too we have made the simplifying assumption that winter will not end at midnight but in the morning after the night that winter ends (which may be 80 ≤ *D* ≤ 100). Period 96 is 8:00 or daybreak any day. For periods with good weather *t* < *T*_f_, the dynamic programming equation becomes:

5 $$F_{\text{G}} (x,y,t) = \mathop {\hbox{max} }\limits_{i} (1 - \beta_{i} )\left[ \begin{aligned} &p_{\text{GG}}^{{}} \left\{ {\lambda_{i} F_{\text{G}} (x^{\prime } ,y^{\prime } ,t + 1) + (1 - \lambda_{i} )F_{\text{G}} (x^{\prime \prime } ,y^{\prime } ,t + 1)} \right\} + \hfill \\ &(1 - p_{\text{GG}} )\left\{ {\lambda_{i} F_{\text{B}} (x^{\prime \prime \prime } ,y^{\prime } ,t + 1) + (1 - \lambda_{i} )F_{\text{B}} (x^{\prime \prime \prime \prime } ,y^{\prime } ,t + 1)} \right\} \hfill \\ \end{aligned} \right],$$

where *i* is behaviour, *λ* is the probability of variation when performing this behaviour (for example, the amount of food found when foraging), and *β* is the instantaneous predation risk. As there are separate functions for good (*F*_G_) and bad weather conditions (*F*_B_, not shown), there will be separate fitness values for good and bad weather conditions. This makes it possible for the bird to optimize behaviour relative to external conditions, i.e., to choose behaviour depending on weather conditions. The variable $p_{\text{GG}}$ is the probability that the current good weather conditions will continue also the next time period. The probability of a change to bad weather conditions will thus be $\left( {1 - p_{\text{GG}} } \right).$ Keeping the transition probabilities for a change in weather conditions $\left( {1 - p_{\text{GG}} } \right)$ and $\left( {1 - p_{\text{BB}} } \right)$ at low values decreases the probability of unrealistic short-term weather fluctuations. We set the baseline value of $p_{\text{GG}}$ to $1 - \frac{1}{2D}$(≈0.9983) and $p_{\text{BB}}$ to $1 - \frac{1}{D}$ (≈0.9965) giving a 61.3% chance that present good weather conditions remain the same over the next 24 h and a 37.5% that it remains the same for the next 48 h. The difference between $p_{\text{GG}}$ and $p_{\text{BB}}$ may seem small, but the probability that weather should remain bad for the next 24 h is 36.4 and 13.3% for the next 48 h. As stated above, the reason for this difference is that we wanted good weather conditions to be more common than bad weather conditions. The four possible values of *X* depend on both weather conditions $\left( {p_{\text{GG}} } \right)$ and foraging success (*λ*).

The thermal state of the animal, *Y*, on the other hand, is affected only by the bird’s decisions whether to change temperature or not. Hypothermia is thus an “active” condition that will change as a function of the bird’s strategic decisions, rather than by ambient temperature variation. For example, the bird can choose to stay non-hypothermic, or enter only shallow hypothermia under cold weather conditions, but will then experience higher energy expenditure compared to if it had entered deeper hypothermia.

Under bad weather conditions, the dynamic programming equation can be written in analogy with Eq. 5, i.e., $F_{\text{B}} (x,y,t)$ with transition probabilities for bad weather $p_{\text{BB}}$ and $p_{\text{BG}} = 1 - p_{\text{BB}} .$ If the part of Eq. 5 in the square brackets is denoted *V*_*i(w)*_, the dynamic programming equation for the periods when winter can end, *T*_f_ ≤ *t* ≤ *T*, becomes:

6 $$F_{w} (x,y,t) = p_{t} \varPhi (x,t + 1) + (1 - p_{t} )\mathop {\hbox{max} }\limits_{i} (1 - \beta_{i} )V_{i(w)} ,$$

where *w* denotes weather conditions that can be either good or bad. The notation with *w* for weather removes the need for a special equation for bad weather conditions, because the transition probabilities for weather conditions can be included in *V*_*i(w)*_.

There are six behaviours (*i*) to optimize over:

1. High-intensity foraging (high reward, high predation risk).
2. Low-intensity (cautious) foraging (lower reward, low predation risk).
3. Rest in predator safe habitat (no reward, minimum predation risk).
4. Decrease body temperature (enter, or go deeper, into hypothermia).
5. Increase body temperature.
6. Stay at constant hypothermic body temperature.

Predation risk for behaviours 4–6 are more complex than for behaviours 1–3. In the baseline version, they are constant and high during daylight, but lower at night (Table 1). Behaviour 1–3 are only possible for non-hypothermic birds (i.e., *Y* = 0), 4 is not possible for birds in the maximal hypothermic condition (*Y* = *Y*_max_), whereas 5 and 6 are only possible for birds that are hypothermic (*Y* > 0). The baseline parameter settings for these behaviours are given in Table 1.

In two of the three previous dynamic programming models on hypothermia, the night has been modelled as a single event with a fixed (Pravosudov and Lucas 2000) or stochastic (Clark and Dukas 2000) energy loss. Welton et al. (2002) modelled nights in a more detailed way. They divided the 24-h day in half-hour periods and allowed behaviour also at night (rest or hypothermic rest). We think that this view on hypothermia is more realistic, but that their model may be too coarse in the sense that body temperature only had two values, hypothermic or non-hypothermic. In the baseline version of our model, we, therefore, have 20 discrete steps for this variable. We assume that the birds can make behavioural decisions both in the day and at night; a hypothermic condition can be entered, changed, or left anytime. As birds of this type will never forage at night, we set the parameter values, so that behaviour 1 and 2 will only occur during daylight hours. In the baseline version of the model, we assume that the birds cannot forage in a hypothermic condition.

The dynamics for the change in body fat deposits *X* under good weather conditions for a non-hypothermic bird will be:

7 $$x^{{ ( {\text{k)}}}} (t + 1) = \left\{ {\left. {\begin{array}{*{20}l} {x + \Delta G_{i} - C_{i} {\text{ with prob }}\lambda p_{\text{GG}} } \\ {x + \Delta G_{i} - C_{i} \gamma {\text{ with prob }}\lambda (1 - p_{\text{GG}} )} \\ {x + \Delta G_{i} \delta - C_{i} {\text{ with prob }}(1 - \lambda )p_{\text{GG}} } \\ {x + \Delta G_{i} \delta - C_{i} \gamma {\text{ with prob }}(1 - \lambda )(1 - p_{\text{GG}} )} \\ \end{array} } \right|y = 0} \right..$$

In general the new level of fat is denoted $(x^{{ ( {\text{k)}}}} )$. For successful foraging under good weather conditions it becomes $x^{\prime}$ (cf. Eq. 5); the second row shows this calculation under bad weather conditions, $x^{\prime\prime},$ etc. *G*_*i*_ is the energy gain from behaviour *I*, which will be 0 for behaviour 3 to 5. ∆ is a factor that will change the energy gain with the same proportion under all conditions. It is set to 1 (no change) under baseline conditions. In many foraging models, (1 − *λ*) will be 0, but in our model, food will still be found, but the gain will be reduced with a factor *δ*. The rationale for this view is that a bird such as a parid normally will find many food items in 5 min. In fact, an intensively foraging parid will find around one food item per minute while feeding nestlings (Gibb 1955) or storing food (Haftorn 1956; Pravosudov 1985; Brodin 1994). While foraging, it will typically peck at small food items such as spiders, aphids, moth cocoons, etc. several times even during 1 min. This makes it unlikely that it would not find any food at all during a 5-min long foraging bout. The amount it finds, on the other hand, will vary stochastically. The baseline value of *δ* is 0.8. If the weather is unsuitable (bad), energy expenditure increases with a factor *γ* with a baseline value of 1.2. *C*_*i*_ is a mass- and activity-dependent energy loss:

8 $$C_{i} = C_{\text{RM}} + \mu_{i} C_{\text{RM}} + \mu_{i} C_{\text{RM}} x/X_{\hbox{max} } ,$$

in which *C*_RM_ is resting metabolism, here defined as the lowest possible metabolism of a resting non-hypothermic bird in winter. The coefficient *µ*_*i*_ is an activity-dependent factor that increases metabolism compared to resting. The baseline value of 2 for activities 1 and 2 gives a metabolic cost of 3× resting metabolic rate for a lean bird that is foraging, whereas the value for resting activities 3–6 is 0 (warming up from hypothermia is of course costly, see below). We have deliberately chosen low values of *µ*, since heat produced by movement substitutes for metabolism when ambient temperature is below thermoneutrality (Pohl and West 1973; Bruinzeel and Piersma 1997; McNamara et al. 2004). The last term is a mass-dependent increase that makes it more expensive to forage for fatter birds. A maximally fat bird would experience a metabolic cost of 5 × *C*_RM_ while foraging.

For a hypothermic bird, the energy expenditure becomes

9 $$\, \left. {x^{{ ( {\text{k)}}}} (t + 1) = x - (C_{\text{RM}} - \varepsilon C_{\text{RM}} (y/Y_{\hbox{max} } ))\gamma_{w} - C_{WUi} } \right|y > 0.$$

In this equation, there is no gain from foraging as we assume that a hypothermic bird cannot forage, not even under daylight conditions. We relaxed this assumption when we investigated the possibility of day-time hypothermia, by assuming that a bird in shallow hypothermia (e.g., with a temperature reduction <2°) can forage according to Eqs. 7 and 10. In Eq. 9, the energy loss is reduced by a fraction *ε* that is multiplied with the depth of hypothermia (*y/Y*_max_). Under baseline settings, *ε* is 0.3, meaning that a bird in maximum hypothermia will save 30% of its resting energy expenditure. It should be noted that the value of *ε* is the factor that matters when it comes to hypothermia. The precise number of degrees of body temperature that this 30% save corresponds to does not affect the model per se; their only purpose is to make illustrations clearer. $\gamma_{w}$ is the same stochastic weather variable as in Eq. 7 with a value of 1.2 for unsuitable weather conditions and 1.0 for good weather. *C*_WUi_, finally, is an extra warming up cost (see Welton et al. 2002). We set this cost to either 0 or 6 kJ (Table 2).

Under bad weather conditions, Eq. 7 is replaced by:

10 $$x^{{ ( {\text{k)}}}} (t + 1) = \left\{ {\left. {\begin{array}{*{20}l} {x + \Delta G_{i} - C_{i} \gamma {\text{ with prob }}\lambda p_{\text{UU}} } \\ {x + \Delta G_{i} - C_{i} {\text{ with prob }}\lambda (1 - p_{\text{UU}} )} \\ {x + \Delta G_{i} \delta - C_{i} \gamma {\text{ with prob }}(1 - \lambda )p_{\text{UU}} } \\ {x + \Delta G_{i} \delta - C_{i} {\text{ with prob }}(1 - \lambda )(1 - p_{\text{UU}} )} \\ \end{array} } \right|y = 0} \right.,$$

with the same symbols as in Eq. 7. The change in *Y*, the degree of hypothermia, is given by the following:

11 $$y^{\prime } = y + \sigma_{\text{i}} - \eta_{\text{i}} ,$$

where $\sigma_{\text{i}}$ is an increase in the hypothermic state (i.e., a decrease in body temperature), and *η* is a decrease in the hypothermic state (i.e., an increase in body temperature). Again, it should be observed that *Y* will have positive values for hypothermic birds, but still depicts a temperature decrease. The increase $\sigma_{\text{i}}$ will be zero for all behaviours, except for behaviour 4 (enter or go deeper into hypothermia). The decrease in hypothermic state is given by:

12 $$\eta_{\text{i}} = \hbox{min} \left\{ {\gamma_{\text{i}} ,y} \right\},$$

where *γ* will be zero for all behaviours except behaviour 5 (increase body temperature). Here, *y* is the current hypothermic state in °C. A body temperature below normal is represented by a positive number, *y*. The min expression makes sure that the birds cannot raise their body temperature more than back to normal. Under baseline settings of parameter values *η* = *σ*, meaning that increases and decreases in body temperature will occur at the same rate.

**Fig. 1:**
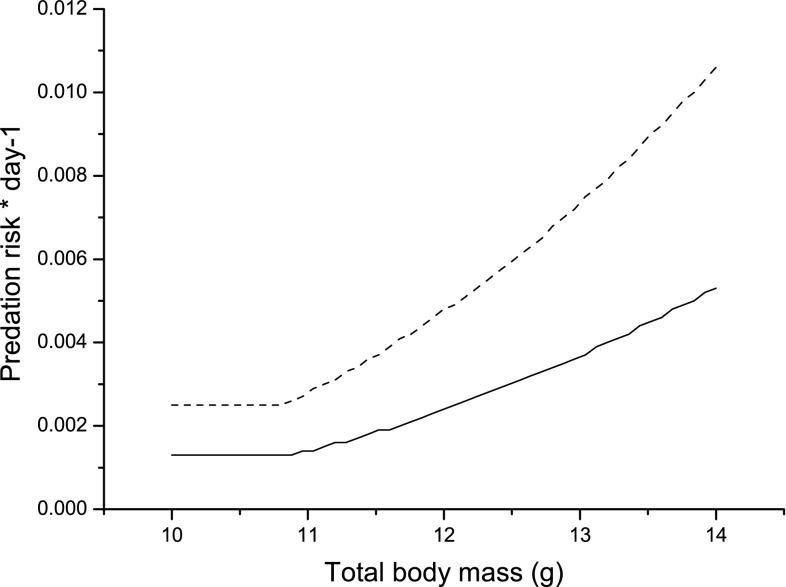
Experienced daily (8-h daylight) predation risk for a bird foraging intensively (behaviour 1, *dashed curve*), or more cautiously (behaviour 2, *solid curve*), under baseline parameter values. We assume that a bird can gain up to 1 g of fat before mass-dependent effects on acceleration and manoeuvrability will have an effect on predation risk (Kullberg 1998; Brodin 2001). Observe that this is a parameter of the model, not a prediction from it

Predation risk is activity- and mass-dependent for non-hypothermic birds during daylight hours:

13 $$\beta_{\text{i}} = \left\{ \begin{array}{lll} \beta_{\text{iB}} + \beta_{\text{iB}} ((x - x_{\text{cr}} )/x_{\text{cr}} )^{\alpha }&\quad {\text{ if }}&x > x_{\text{cr}} \\ \beta_{\text{iB}}&\quad {\text{ if }}&x \le x_{\text{cr}} \\ \end{array} \right.,$$

where, $\beta_{\text{iB}}$ is the basic activity-dependent predation risk for a lean bird, *x* is the present body mass, and *x*_cr_ is a critical limit body mass above which predation risk becomes mass-dependent in an accelerating way with *α* being the exponent for this increase (cf. Brodin 2001). The day-time predation risk experienced, while foraging is shown in Fig. 1. Under baseline conditions, we assumed that the predation risk for hypothermic birds, *β*_h_ was high and constant during daylight hours under the assumption that a hypothermic bird in daylight would experience a high predation risk if spotted by a predator.

Recent empirical studies suggest that birds could be in shallow day-time hypothermia when they forage, and that they are able to fly in this state (Carr and Lima 2013; Lewden et al. 2014). To investigate under what conditions this could occur, we reduced the predation risk for day-time hypothermia and, subsequently, relaxed the assumption that such birds cannot forage. We first assumed that day-time predation risk should increase linearly with a decrease in body temperature:

14 $$\beta_{\text{h(day)}} = \beta_{3} + (y/Y_{\hbox{max} } )\beta_{\text{h(max)}} ,$$

where *β*_3_ is the mass-dependent predation risk for behaviour 3 in Eq. 13 (resting in a predator safe habitat), *y* is the decrease in body temperature in C, *Y*_max_ is maximum hypothermia, and *β*_h(max)_ is the high baseline predation risk during daylight hours for hypothermic birds. Day-time predation risk will then increase from its minimum (non-hypothermic lean bird) to its maximum (maximally hypothermic fat bird), as a function of both the decrease in body temperature and the increase in body mass. As this was not sufficient to create day-time hypothermia, we continued by setting day-time predation risk for hypothermic birds to be as low as for behaviour 3 (resting in safe habitat), but this was neither sufficient to create day-time hypothermia. Our next step was to remove the assumption that hypothermic birds cannot forage during daylight hours (cf. Eq. 9), by assuming that there was some limit (e.g., 2 °C) below which they could not forage. Above that limit, we assumed that they could forage in the same way as normothermic birds. This can be implemented by changing the |*y* = 0 to |*y* < 2 on the right side of Eqs. 7 and 10.

A dynamic programming equation iterates backwards in time, starting from the last possible day of winter and ending in the morning the first day of winter. Such a calculation produces the optimal behaviour at each time step for all possible states of a bird, including states that an optimally behaving bird will never reach, e.g., unrealistically high fat levels. In the forward iteration, we simulated 1000 birds with a morning body mass of 11.2 g on day 1 (which was the preferred level when the iteration had stabilized after a few days) in a normothermic condition. These “virtual birds” used the optimal behaviour that was calculated with the dynamic programming equations. The outcome is stochastic, and the mean will produce curves such as those in Figs. 3 and 4.

## Results

**Fig. 2:**
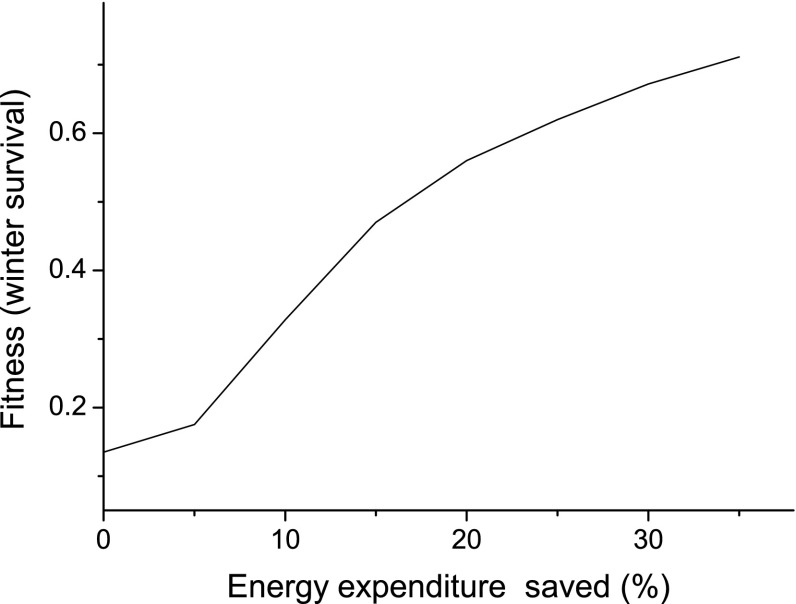
Survival probability (fitness) over the whole winter as a function of the maximum reduction in energy expenditure during nocturnal hypothermia

The probability of winter survival increased dramatically from 0.13 to 0.71 if birds used hypothermia to save 30% of the overnight energy expenditure (Fig. 2). A saving of 5% would increase survival only slightly, but above this value, the fitness gain increased rapidly. The increase was strongest for energy saves between 5 and 15%, after which it levelled off somewhat for additional 5% steps (Fig. 2).

**Fig. 3:**
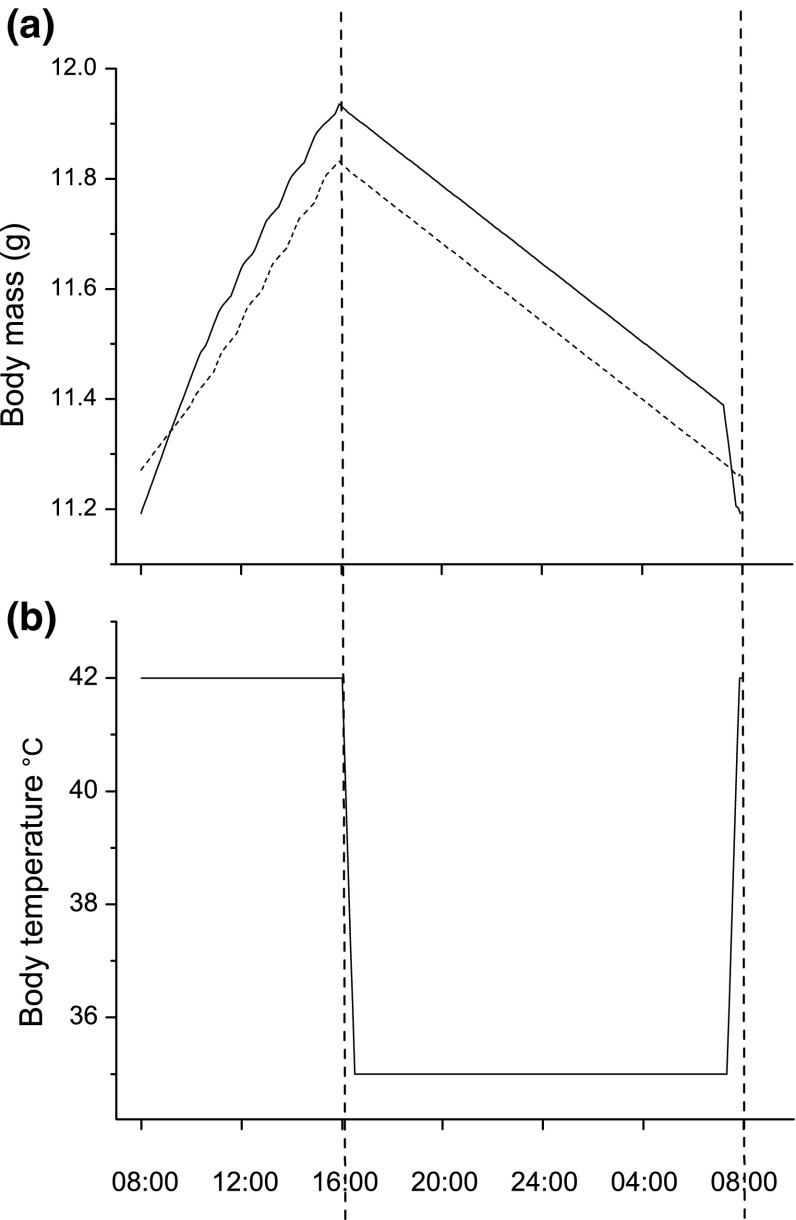
Optimal body mass (**a**) and body temperature (**b**) trajectories a day in mid-winter if the maximum hypothermic save is 30%. The *dotted*, *vertical*, *lines* show the beginning and end of the night, respectively. In **a,** there is no extra warming up cost for the *dashed curve* and 6 kJ extra warming up cost for the *solid curve*. The *curves* show the means for 1000 “individuals” in the forward iteration. *No dispersion bars* are shown as the standard deviation is almost symmetric below 0.03 for all data points in both curves. To include such *small error bars* would have no other effect than to make the *curves* appear *very thick* and *blurry*, thereby decreasing the clarity of the figure

From here on, we will use the term “should” to describe a bird using the optimal behaviour predicted by the model. As lean body mass is constant in the model, we will refer to changes in the body mass curve as fat gain or fat loss. The body mass and body temperature trajectories under baseline settings of parameter values could have been taken from a field study of small parids in a cold winter forest (Ekman and Lilliendahl 1992; Reinertsen and Haftorn 1983, Fig. 3). In the morning, birds should forage intensively with a normothermic body temperature, 42 °C (behaviour 1). They should keep on foraging all daylight hours until dusk (Fig. 3a). After noon, the body mass curve shows a slightly uneven pattern. This is explained by the fact that as the probability that the birds will reach the optimal fat level at dusk increases, they will start to use the second foraging strategy, cautious foraging with less gain (behaviour 2). Depending on the foraging success in the afternoon, the birds will switch between behaviours 1 and 2 to reduce the high predation risk of behaviour 1. The total fat gain over the whole day will be 0.74 g, a typical amount for a willow tit, blue tit, or black-capped chickadee in winter (Lilliendahl 2002). The birds should immediately start decreasing body temperatures when it gets dark, until they reach the minimum body temperature (behaviour 4, Fig. 3b). According to Reinertsen and Haftorn (1986), willow tits always use hypothermia at night under cold conditions, and in winter, their body temperatures will fall quickly in the evening and increase quickly in the morning (Reinertsen and Haftorn 1983). The birds should remain in this hypothermic state the whole night (behaviour 5), but start increasing body temperature around 30 min before dawn (behaviour 6), so that they are back to normal body temperature when daylight arrives. Adopting this strategy minimizes the risk of being spotted by a predator in a lethargic condition. If there is an extra warming up cost of 6 kJ, the rate of fat metabolism increases (the rightmost part of the curve in Fig. 3a), because the birds have to burn fat at a high rate to increase body temperature. If there is such a cost, the birds would carry 0.1 g extra fat already at dusk to buffer for this expense (Fig. 3a).

**Fig. 4:**
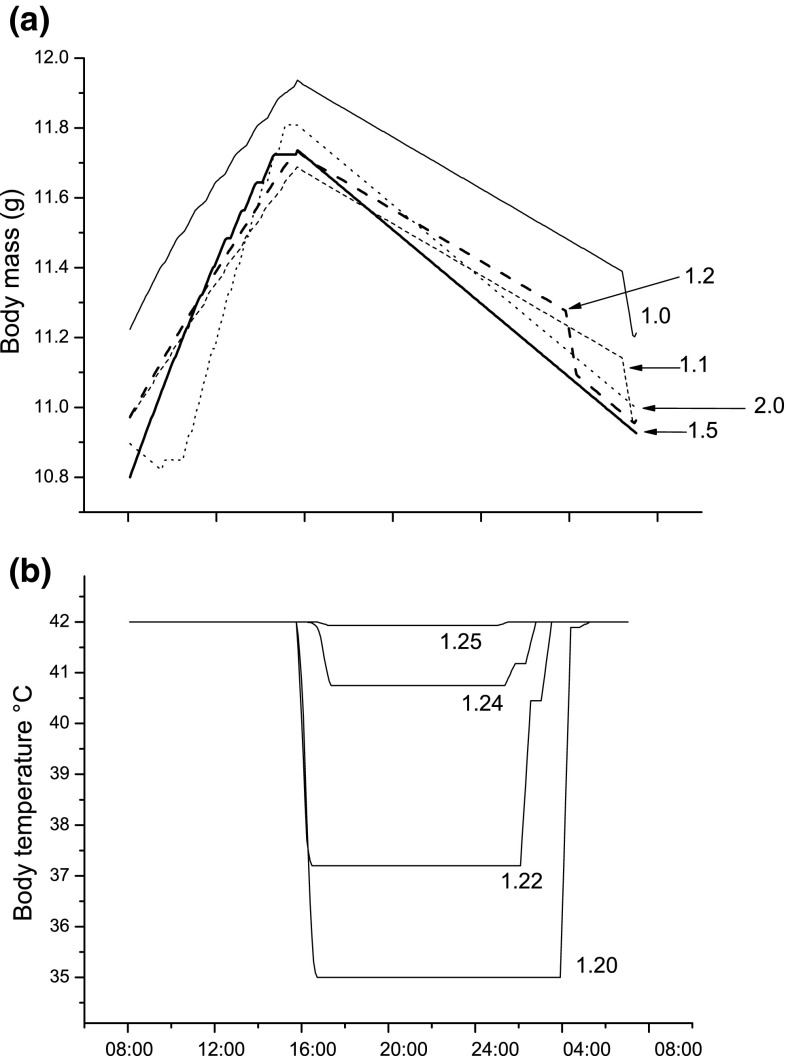
Optimal body mass (**a**) and body temperature (**b**) trajectories under various food availability regimes, i.e., various values of *∆.* This factor gives the same proportional increase of food availability under both good and bad weather conditions. For example, *∆* = 1.2, which means that food availability has increased with 20% compared to baseline conditions. The *curves* are produced in the same way as Fig. 3 in the forward iteration

Figure 3 is made under the assumption that the maximum energy saving from hypothermia is 30% of resting metabolism, but it is possible that it may be considerably smaller under cold conditions (Reinertsen and Haftorn 1986; Cooper and Gessaman 2005). A gradual decrease of the maximum energy saving reveals that the body temperature curve (cf. Fig. 3b) would remain identical until the save is only 4%, i.e., when night-time hypothermia should be abandoned completely (not shown). The body mass curve (Fig. 4a) would remain almost identical in shape but shift slightly downward with a decreasing saving. When hypothermia is abandoned at around 4% savings, the curve would shift almost 1 g upwards as the birds need considerably more fat for the night (not shown).

The birds should never rest (behaviour 3) during daylight hours (Fig. 3), but only switch between the two modes of foraging using safe foraging (behaviour 3) whenever this is possible. This may well describe the reality for a small bird on a cold winter day in the boreal zone. To investigate how birds should behave under less severe conditions, we decreased the energetic stress by gradually increasing the gain from foraging. For example, a *∆* of 1.2 (i.e., a 20% increase in gain rate) would change the gain from intensive foraging from 80 to 96 kJ, and for low-intensity foraging from 60 to 72 kJ if all eight daylight hours are spent foraging. Increasing *∆* could possibly correspond to conditions with human-provided supplemental feeding or those with a higher natural food abundance, such as in autumn.

As *∆* increases to 1.1 (i.e., a 10% higher gain rate for both types of foraging), the birds should reduce the amount of fat carried by approximately 0.2 g (Fig. 4a, dotted curve). The effect will essentially be that the body mass curve is shifted downwards, but with retained shape. A further increase to 1.2 (i.e., a 20% higher gain rate) will shift the curve slightly upwards again, but now a qualitative difference appears (Fig. 4a, dashed curve). Energy expenditure increases rapidly when the birds leave the hypothermic state already around 4 h before dawn (Fig. 4b, bottom curve). The use of hypothermia at night disappears in a narrow window between *∆* of 1.2–1.3 (Fig. 4b). When food availability increases, the birds should gradually decrease high-intensity foraging (behaviour 1) as the gain rate from cautious foraging (behaviour 2) becomes sufficient. At *∆* over 1.2, the gain rate from cautious foraging will be higher than that from intensive foraging under baseline conditions. The unevenness of the *∆* = 1.5 curve in the afternoon depends on the birds switching between safe foraging and resting (behaviour 3). Under conditions when the gain rate is very high (*∆* = 2, Fig. 4a dotted curve), the energetic situation is now so relaxed that birds can rest in the morning before they start to forage around 11 o’clock.

If a bird becomes hypothermic during daylight hours, it should immediately increase its body temperature and leave this condition (not shown). If it is only in shallow hypothermia (or normothermic) at night, it should decrease its temperature to maximum hypothermia (not shown). In the baseline version of our model, we assumed that predation risk was very high for hypothermic birds during daylight hours. The reduction of predation risk according to Eq. 14 makes shallow hypothermia to a relatively safe strategy, but it still does not occur during daylight hours. Not even when day-time predation risk for hypothermic birds was as low as for birds resting in a safe habitat (behaviour 3) would the birds ever use facultative hypothermia during daylight hours. Nor would a relaxation of the energetic stress create conditions resulting in the use of day-time hypothermia (Fig. 4b), not even when combined with low day-time predation risk for hypothermic birds. On the other hand, if we allowed the birds to forage in shallow hypothermia, this became the optimal strategy (not shown). If, for example, we assumed that birds could forage normally down to 2 °C below normal body temperature, such a shallow hypothermia would be optimal.

**Fig. 5:**
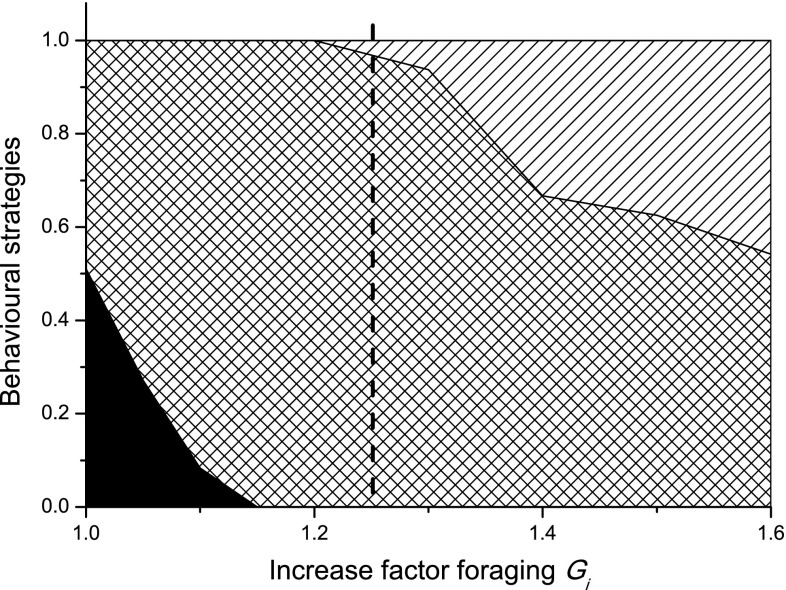
Optimal behavioural strategies as a function of food availability plotted as proportion of the day spent on an activity. On the *x*-axis, 1.0 is the baseline value of ∆. The plot shows the proportion of the day spent on high intensive foraging (behaviour 1; *black*), low-intensity foraging (behaviour 2; *crossed*) and resting in safety (behaviour 3; *diagonally hatched*). The dotted vertical line shows the *G*_*i*_ value when night-time hypothermia is completely abandoned

 In Fig. 5 the proportion of daylight time the birds should spend on behaviours 1–3 under various levels of food availability are shown, together with the level of increased gain in foraging when they should abandon the use of night-time hypothermia.

## Discussion

The most important message from this model is that night-time hypothermia that is frequently used by many northern passerines may increase winter survival by almost 60%. Even if we assume that the maximum saving is less than 30%, as could be likely in low ambient temperature (Reinertsen and Haftorn 1986; Cooper and Gessaman 2005), the effect would be huge. For example, a maximum of 15% energy savings would increase survival by 33.5%. In winter, parids typically roost in cavities, which decrease heat loss considerably. Below environmental temperatures of −10 °C, they will frequently sleep in snow cavities (Haftorn 1972; Novikov 1972; Helle 1980; Korhonen 1981), where temperatures might even be quite warm during cold winter nights (Korhonen 1981). Under such conditions, i.e., when the need for metabolic heat production is much reduced, the savings accrued from facultative hypothermia may be closer to 30% than to 15%.

We believe that we have succeeded in creating a realistic model with appropriate settings of parameters for a typical “little bird in winter”. This is supported by the curves in Fig. 2 that shows very realistic fat and body temperature trajectories of a small passerine such as a parid under cold winter conditions. We are aware that the pattern in body temperature as depicted in Fig. 2 may over-exaggerate patterns seen in empirical measurements (e.g., Reinertsen and Haftorn 1983, 1986; Cooper and Gessaman 2005). This simplification was in part necessary to make the model manageable (and still represent a vast increase in resolution relative to the previous, similar, models; above), and in part a consequence of the instant changes in environmental conditions at dusk/dawn in our model. It is important to note that the shape of the body temperature curve does not affect inferences in any way, because the parameter of interest in the model is always overnight energy expenditure. In mid-winter, species such as willow tits, Siberian tits, boreal chickadees, and black-capped chickadees will carry larger fat reserves than at other times of the year, spend essentially all daylight hours foraging, and enter a lethargic hypothermic state when it gets dark (Reinertsen and Haftorn 1986; Brodin unpublished data). If it gets cold in Southern Sweden, also blue tits will follow similar trajectories (Nord et al. 2009). It could be argued that our model species, the blue tit, may not be as well adapted to cold winter conditions as for example the willow tit, the Siberian tit, or the boreal chickadee. Still, we think that our model demonstrates the significance of facultative night-time hypothermia for winter survival in small birds. The above-mentioned parid species (that all spend the winter in the boreal coniferous forest) are all large-scale food hoarders. A bird that can combine the benefits from stored food supplies with an ability to enter hypothermia at night will be well adapted to the harsh environment in a boreal coniferous forest in winter.

We had difficulties in creating conditions when birds would choose to enter hypothermia during daylight hours. Clearly, the key to this was whether hypothermic birds could forage or not. Otherwise, under conditions when hypothermia would be required to conserve energy, birds cannot use this strategy, since all daylight hours are needed for foraging. Under more relaxed conditions, when birds do not have to forage the whole day, hypothermia is no longer needed. It should be noted that if we allowed birds in shallow hypothermia to forage, all birds would always choose this strategy. Even if day-time hypothermia does occur in parids (Lewden et al. 2014, 2017), it is, as far as we know, a rare phenomenon. This suggests that the strategy is associated with some costs, such as reduced foraging ability or increased predation risk (cf. Carr and Lima 2013). Birds that nevertheless adopt in this condition may have been affected by some unexpected event such as illness or other types of energetic emergencies.

The shifts of the fat gain/loss and body temperature curves (Fig. 4) with increasing gain rate have some interesting implications for the phenomenon known as winter fattening. An increase of the food availability with 10% (i.e., from *∆* = 1 to 1.1, Fig. 4a) moved the gain curve downwards, but with the same shape and slope of the energy gain rate. The birds cannot choose to gain energy faster or slower, to rest, or to leave hypothermia at night, etc. Even though the environment becomes less harsh, the optimal behaviour still seems to be regulated by the environmental harshness. True winter fattening means that birds not only gain more fat before the night, but that they also carry larger morning reserves (Lehikoinen 1987). This is essentially illustrated by the difference between the two curves in Fig. 4a.

As gain rate increases more than 10% (i.e., above *∆* = 1.1), the birds can afford to make strategic decisions. For example, they can chose to decrease the use of hypothermia at night, spend some daylight time resting, and choose to gain energy at various rates, etc. In analogy with Verhulst and Hogstad (1996), this can be viewed as the birds gradually gaining more strategic control over their energy management. Under baseline conditions (*∆* = 1), energy management can be viewed to be under stronger environmental control. In agreement with the previous models of facultative hypothermia in small passerines (Clark and Dukas 2000; Pravosudov and Lucas 2000; Welton et al. 2002), we have assumed that the main cost of hypothermia is an increase in predation risk. The large impact of predation risk in our model is shown by the rapid abandonment of hypothermia when gain rate increases (Fig. 4b). There is empirical support for this, for our model, species, because blue tits that enter nocturnal hypothermia in nature will abandon this strategy when they are given ad libitum access to food (Nord et al. 2009). In addition, they will reduce the depth of hypothermia in the wild as a response to an increase in the fat deposits that they carry (Nord et al. 2011). Hypothermia may increase predation risk also at night, since small parids tend to sleep in nest boxes or tree or snow cavities in winter. Predators such as mustelids and feral cats can then attack sleeping birds in their cavities. A hypothermic bird may then be slow or unable to react, whereas a non-hypothermic bird may be quicker to escape.

Under almost all conditions (except for a narrow window when 1.22 < *∆* < 1.25), the birds should always enter maximum hypothermia at dusk and stay in this condition for as long as it is needed. Under conditions when hypothermia is only needed for a part of the night, the rest in normothermia should always follow after the hypothermic period. The reason is that temperature varies stochastically and the closer the morning gets, the higher the probability that the bird will reach its morning goal mass. This implies that a bird that starts the night with normal body temperature and subsequently enters hypothermia later at night has experienced some sort of unexpected energetic emergency.

A gradual increase of the energy gain from foraging gave the prediction that birds should abandon the use of hypothermia at fat levels when they still cannot afford to rest during the day (Fig. 5). This suggests that avoiding hypothermia should be more important than to minimize day-time predation risk by resting in predator safe locations, a notion well in line with the observation that small birds consistently seem to maintain the highest affordable night-time body temperature (see Nord et al. 2009, and references therein). Apart from reducing predation risk, avoidance of hypothermia might also be beneficial for minimizing potential physiological costs of hypothermic body temperatures, such as impaired immune function (Nord et al. 2013; Sköld-Chiriac et al. 2015) and reduced quality of sleep (Mueller et al. 2012; Deboer and Tobler 1996). High-quality sleep may be important for these kinds of birds, since it can facilitate memory retention and somatic repair, thereby increasing the likelihood of retrieving cached food (Roth et al. 2010). In conclusion, our model shows that energy saving by nocturnal hypothermia is crucial for winter survival for small passerines even if they can only save 10–30% of the overnight energy expenditure. Our model is well designed and parametrized, as its predictions fits very well with empirical data on behavioural and physiological responses of small birds such as parids under cold winter conditions. The model illustrates the mechanisms behind the phenomenon known as winter fattening and shows that facultative hypothermia in the day should not be a common (at least if this strategy constrains foraging ability and/or gain rate). Finally, in an unpredictable environment, birds should always hedge against stochasticity by maximizing energy saving during the first part of the night, and energy gain in the early parts of the day. Later, when the probability that they will reach their goal fat levels, they can behave more cautiously, e.g., by leaving hypothermia at night or forage more cautiously in the day.

## Electronic supplementary material

Below is the link to the electronic supplementary material.

Supplementary material 1 (doc 20 kb)
