# Supplementary material for: Adaptive temperature regulation in the little bird in winter: predictions from a stochastic dynamic programming model
Source: Oecologia. 2017 Aug 3;185(1):43–54. doi: 10.1007/s00442-017-3923-3 (PMC5596050; doi:10.1007/s00442-017-3923-3)
Supplement: Supplementary file 1 — Supplementary material 1 (doc 20 kb) [file 442_2017_3923_MOESM1_ESM.docx]

**Appendix 1** **– Justification of parameter values**

We used parameter values from willow tits, because winter energy expenditure is better known in this species than in blue tits. We are confident that estimates for willow tits are relevant also for blue tits, because these two species are closely related and very similar in body mass (± 1 g). The mean daily energy expenditure (i.e., field metabolic rate, FMR, over 24 h) in a willow tit is around 45 kJ d^-1^ in winter (Moreno et al. 1988) if it forages for eight hours and sleeps for 16 hours (during which time it is likely hypothermic [cf. Reinertsen and Haftorn 1983] and, thus, have substantially reduced FMR). It is hard to precisely estimate the daily energy expenditure for a *normothermic* bird if it instead rested for 24 hours: on the one hand a resting bird may spend less energy than an actively foraging bird (if heat substitution from activity is only modest; cf. Paladino and King 1984), but on the other hand this bird does not accrue any energy savings from nocturnal rest-phase hypothermia. For simplicity, we will therefore assume that the daily energy expenditure for a resting, normothermic, blue tit is similar to FMR in willow tits, i.e.45 kJ d^-1^. During active foraging, we assume that FMR is two times higher than resting metabolism (Moreno et al. 1988).

The energy gain from intensive foraging (‘Behaviour 1’) should be substantially higher than daily energy expenditure if the bird is to remain in energy balance. Hence, we set the gross gain from high intensity foraging (‘Behaviour 1’) to 80 kJ for a whole eight-hour day under good weather conditions. As energy expenditure is mass-dependent (equation 8), the net energy gain will be 35 to 50 kJ depending on the body mass of the bird. Cautious foraging (‘Behaviour 2’) yields a gross gain of 60 kJ for a full foraging day, with a net gain of 15 to 30 kJ depending on body mass. One gram of fat contains 37 kJ (Withers 1992), meaning that a bird in our model could deposit 0.95 to 1.35 g of body fat reserves (depending on body mass) by foraging intensively under good weather conditions, or 0.41 to 0.81 g by foraging cautiously, if it feeds actively for the whole day. We set the maximum energy savings from rest-phase hypothermia s to 30% of resting metabolism, and consider this to correspond to a 7°C decrease in body temperature (as observed in willow tits by Haftorn (1972), and Reinertsen and Haftorn (1983)).

We consider predation risk to be mass-dependent and let the shape of the curve follow a theoretical model based on empirical measurements (Brodin 2001). Hence, a bird carrying less than 1 g of fat would have a 60.6% probability to avoid death from predation if all 9600 daylight periods of the winter are spent on high-intensity foraging (‘Behaviour 1’). If all daylight periods instead are spent foraging cautiously (i.e., ‘Behaviour 2’), the survival probability would increase to 88.2%. We set a high baseline predation risk for daytime hypothermia: birds using hypothermia under daylight conditions had a 5% risk of getting killed by a predator in an eight–hour day. Consequently, a bird that is hypothermic during all daylight periods would have less than 1% chance of avoiding predators the whole winter. The main predator of willow tits in Scandinavia in winter is the pygmy owl *Glaucidium passerinum*, which is diurnal during this time of the year (Ekman and Askenmo 1986). It follows that daytime predation is more common than night-time predation in this system. Thus, we set a relatively low night-time predation risk for a hypothermic bird, viz. 3.7 $\times$ 10^-4^ for a 16 hour night. This gives a bird that uses rest-phase hypothermia a 96.4% probability of avoiding getting killed by a nocturnal predator over the whole winter.

The cooling (*σ*) and warming up (*η*) rates are set equal to *Y_max_*/6 per period meaning that the maximum body temperature change (7 °C) will take 30 minutes. Welton et al. (2002) suggest that an additional warming up cost might be important, and include this as a time cost (because foraging time is lost). Here, we instead include an extra energetic cost *C_WUi_* of warming (equation 9). For simplicity we set this cost to a fixed value between 0 to 6 kJ. .

**References**

Brodin A (2001) Mass-dependent predation and metabolic expenditure in wintering birds: is

there a trade-off between different forms of predation? Anim Behav 62: 993-999

Ekman J, Askenmo C (1986) Reproductive cost, age-specific survival and a comparison of the reproductive strategy in two European tits (genus *Parus*). Evolution 40: 159-168

Haftorn S (1972) Hypothermia of tits in the Arctic winter. Orn Scand 3: 153-166

Moreno J, Carlson A, Alatalo RV (1988) Winter energetics of coniferous forest tits Paridae in the north: the implications of body size. Funct Ecol 2: 163-170

Paladino FV, King JR (1984) Thermoregulation and oxygen consumption during terrestrial locomotion by white-crowned sparrows *Zonotrichia leucophrys gambelii.* Physiol Zool 57: 226-236

Reinertsen RE, Haftorn S (1983) Nocturnal hypothermia and metabolism in the willow tit *Parus montanus* at 63 degrees N. J Comp Physiol B 151: 109-118

Welton NJ, Houston AJ, Ekman J, McNamara JM (2002) A dynamic model of hypothermia as an adaptive response by small birds to winter conditions. Acta Biotheor 50: 39-56

Withers PC (1992) Comparative Animal Physiology. Fort Worth: Saunders College
